# How do I do it? Laparoscopic extraperitoneal reverse puncture technique for distal catheter placement in ventriculoperitoneal shunting

**DOI:** 10.1007/s00701-026-06959-4

**Published:** 2026-06-20

**Authors:** ZhuoYing Du, XiaoFei Zhang, ShuangJie Wu, Qiang Yuan, YiFan Tang

**Affiliations:** 1 Department of Neurosurgery, Huashan Hospital, Fudan University, No. 958, Jing Guang Road, Minhang District, Shanghai, 201100 China; 2 National Center for Neurological Disorders, Shanghai, China; 3 Neurosurgical Institute of Fudan University, Shanghai, China; 4 Department of General Surgery, Huashan Hospital, Fudan University, Shanghai, China

**Keywords:** Ventriculoperitoneal shunt, Distal catheter migration, Laparoscopic surgery

## Abstract

**Background:**

Distal catheter migration and obstruction remain a significant cause of shunt failure after ventriculoperitoneal shunting (VPS). A fast and reliable distal catheter fixation can be achieved via a simple laparoscopic extraperitoneal reverse puncture (LERP) technique with minimum complications.

**Method:**

In this technical note, we introduce the workflow of the LERP technique for a safe and reliable distal catheter fixation.

**Conclusion:**

The LERP technique can provide safe and reliable distal catheter fixation and may help reduce distal catheter migration related complications in VPS.

**Supplementary Information:**

The online version contains supplementary material available at 10.1007/s00701-026-06959-4.

## Introduction

Ventriculoperitoneal shunting (VPS) is the mainstay treatment for hydrocephalus [3]. Nevertheless, shunt failure remains common, with an incidence of up to 40% within 2 years postoperatively [5]. Distal catheter obstruction, including catheter migration and omental wrapping, accounts for a substantial proportion of shunt failures [6]. Given the absence of omentum, the suprahepatic space has long been recognized as an optimal location for distal catheter placement [4, 9]. Various techniques have been described for catheter positioning and fixation [2, 8, 10]. Here, we introduce the laparoscopic extraperitoneal reverse puncture (LERP) technique, which allows safe and effective placement and secure fixation of the distal catheter in the suprahepatic space.

## Relevant surgical anatomy

- 1) Suprahepatic space within the peritoneal cavity.

The suprahepatic space (i.e. subphrenic space) is located between the inferior surface of diaphragm and the superior surface of the liver. It is divided into the right and left compartment by the falciform ligament. It is often considered ideal for distal catheter placement due to minimum risk of omentum wrapping.

- 2) Diaphragmatic extraperitoneal space.

This space is the top-most part of the extraperitoneal space, which sits right above the suprahepatic space separated by the diaphragmatic peritoneum. Its avascular nature allows a safe entry for distal catheter during the VPS surgery.

## Description of the technique

### Patient position and preparation

The procedure was co-performed by one neurosurgeon and one general surgeon in a standard neurosurgical operating room. Electromagnetic neuro-navigation system (AxiEM, Medtronic, inc.) and laparoscopic equipment were prepared. All patients received general anesthesia and were placed in supine position with head slightly rotated to the contralateral side of the shunt. The operating table was tilted 15° in the reverse Trendelenburg position to facilitate exposure of the suprahepatic space. Surgical areas were thoroughly sterilized and carefully draped.

### Ventricular entry and subcutaneous tunneling (Neurosurgeon)

The Kocher’s point was used for lateral ventricle entry. A curved incision was made and a burr hole was created underneath the scalp flap. Then the dura mater, arachnoid membrane and cortex were opened step by step. Any hemorrhage was coagulated using a bipolar instrument. The ventricular catheter was introduced under neuro-navigation. Metzenbaun scissors were used to create the scalp tunnel for the accommodation of the shunt valve. The subcutaneous tunnel was further extended using a steel rod to connect the cranial and the abdominal incisions. The ventricular catheter, shunt valve and distal catheter were properly connected and threaded through the tunnel. Then, the distal catheter was externalized through a 0.5 cm incision which was made 2–3 cm inferior to the xiphoid process. The externalized catheter was further trimmed to an appropriate length, with the distance from the incision to the catheter tip measuring approximately 15–20 cm. The catheter was then carefully dressed before finally introduced into the peritoneal cavity.

### Extraperitoneal reverse puncture and intra-abdominal catheter placement (General Surgeon)

**Fig. 1:**
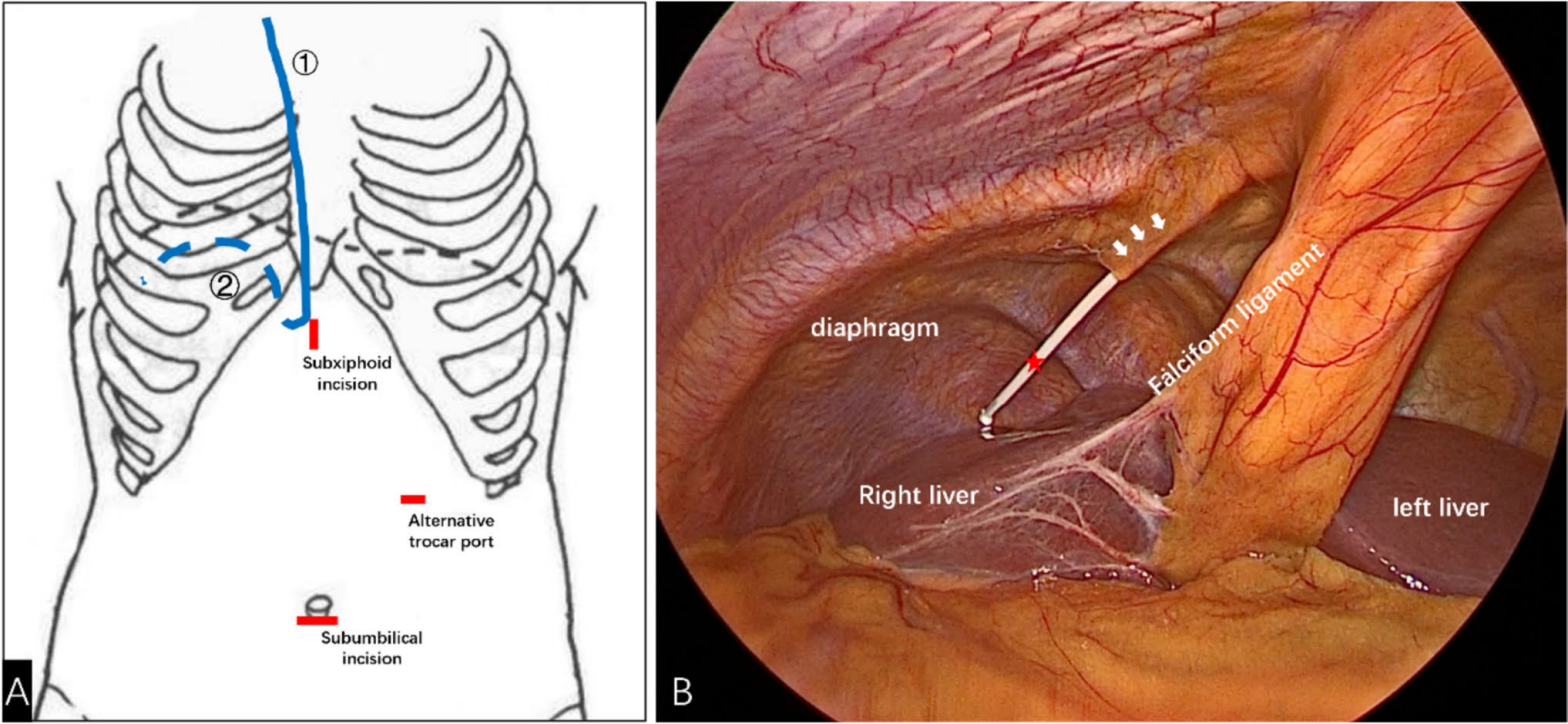
**A** Illustrating abdominal incisions and overall route of the distal catheter. Note that an alternative left upper abdominal trocar port could be deployed when extra surgical maneuver is needed. ① Subcutaneous tunnel. ② Extraperitoneal reverse tunnel. **B** Illustrating the catheter (red star) traveled inside the extraperitoneal reverse tunnel (white arrow) towards the diaphragmatic dome where it entered the peritoneal cavity

The abdominal cavity was entered under direct vision via a 1.5 cm curved subumbilical incision. A 10 mm Trocar was placed, and pneumoperitoneum was established with an intra-abdominal pressure of 12–14 mmHg. The laparoscope was introduced and a careful inspection of the abdominal cavity was performed to ensure a safe placement of the catheter (Fig. 1).

**Fig. 2:**
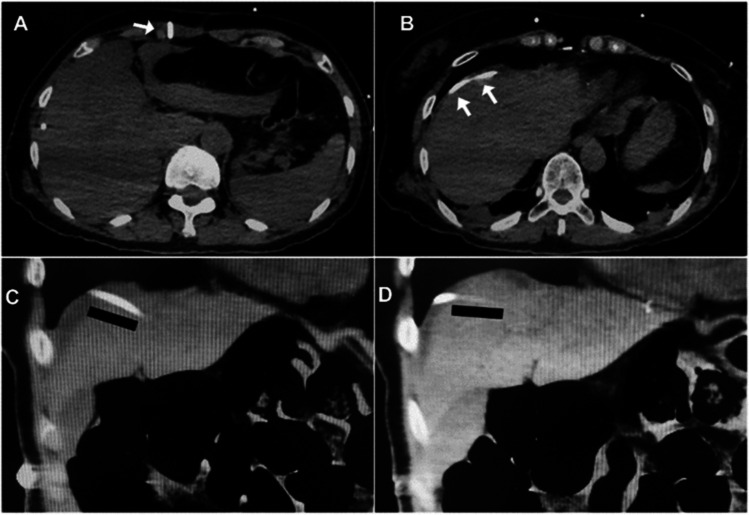
Postoperative CT shows the position of the distal catheter. **A** axial CT image illustrating the site (white arrow) where the catheter entered the extraperitoneal tunnel. **B** the position of the distal catheter in the suprahepatic space (white arrow). **C**, **D** Coronary reconstructed CT image showing the catheter secured inside the suprahepatic space (black bar)

The extraperitoneal tunnel was further created using blunt puncture. The distal catheter was loosely attached to the tip of the steel rod with a silk suture loop. The rod carrying the catheter was inserted through the subxiphoid incision, traversed the rectus sheath to enter the extraperitoneal space, and was reversely advanced along this space toward the diaphragmatic dome before penetrating the peritoneum into the abdominal cavity. As the rod penetrated the peritoneum, the catheter was simultaneously guided into the cavity. The rod was then withdrawn, leaving the catheter mobile within the tunnel (Fig. 1). After confirming patency, the remaining cathXeter was advanced along the tunnel and securely positioned in the suprahepatic space (Fig. 2).

The surgical team then confirmed the position and patency of the shunt system one last time and all incisions were closed.

## Indication

The LERP technique is indicated for patients undergoing conventional VPS surgery, particularly those with a high risk of omental wrapping or unwanted migration.

## Limitations

The procedure requires laparoscopic equipment and a multidisciplinary surgical team with experience in both neurosurgery and laparoscopic surgery, which may limit its application in centers without relevant resources. It is also prohibited for patients in whom suprahepatic cannot be properly visualized, e.g. extensive intra-abdominal adhesion from previous surgeries.

## How to avoid complications?

1. Careful preoperative evaluation concerning the abdominal condition should be carried out, particularly the history of previous abdominal surgeries.
2. Intraoperative aseptic rules should be strictly applied throughout the procedure to prevent shunt contamination.
3. During extraperitoneal reverse puncture and entry of the peritoneum, direct laparoscopic visualization is vital to ensure safe passage and accurate puncture. Care should be taken to avoid liver and diaphragm injury.
4. It is recommended that the steel rod be custom-made to a smaller profile to minimize the injury during the tunneling.
5. The connection between the catheter and the shunt valve should be securely fixed with non-absorbable sutures to prevent catheter disconnection or leakage, and the patency of the entire shunt system must be verified before closure.
6. The direction and position of the distal catheter in the suprahepatic space should be confirmed under laparoscopy to avoid catheter kinking or twisting, which may lead to shunt dysfunction.
7. Routine postoperative care is generally adequate, however, close vigilance for any abnormalities is preferred.

## Specific information for the patient

While the ventricular entry and placement of the proximate part of the shunting system remains the same with traditional VPS, LERP technique provides several benefits. The cooperation within the multidisciplinary team guarantees maximum efficiency and safety during the placement of the shunt system, particularly the distal catheter under direct laparoscopic vision [7]. Featuring part of the distal catheter routing within the reversed upward extraperitoneal tunnel, the LERP technique naturally provides a reliable fixation and minimizes the risk of unexpected catheter migration without any extra intra-abdominal surgical maneuver. A tailored length of the catheter ensures the tip of the catheter resides in the suprahepatic space, minimizing the risks of omentum wrapping or migration.

However, complications such as hemorrhage, parenchymal injury, infection and obstruction remain possible. Laparoscopic approach boasts favorable safety, posing no higher risks than conventional open laparotomy. Still, rare complications including intestinal, diaphragmatic and abdominal visceral injuries may occasionally occur [1]. Some patients might experience brief pain around the diaphragmatic area. If the patients present with deterioration of neurological function or abdominal symptoms, they should revisit the hospital as soon as possible.

The laparoscopic surgery has an extra charge for the equipment used, which is fully reimbursed by the public medical insurance.

## Key points

i. The procedure is co-performed by a neurosurgeon and a general surgeon to ensure maximum safety and efficiency.
ii. The technique utilizes the diaphragmatic extraperitoneal space, an avascular area between the diaphragm and the liver, providing a safe entry route.
iii. The reverse Trendelenburg position is adopted to facilitate better exposure of the suprahepatic space.
iv. Optimal position of the ventricular catheter is essential for avoiding shunt failure. Electromagnetic neuro-navigation provides maximum precision and flexibility as it does not need a rigid head fixation.
v. The subcutaneous tunnel should be made both adequately and gently to keep the shunt system from over-stretched while minimizing the wound pain after the surgery.
vi. By routing the catheter through a reversed upward extraperitoneal tunnel, the LERP technique provides natural, secure fixation and prevents migration without requiring complex intra-abdominal surgical maneuvers.
vii. The length of the catheter from subxiphoid incision to tip is trimmed to 15–20 cm, which effectively limits the mobility of the catheter tip and reduce the risk of long term migration.
viii. The laparoscopic visualization is crucial during the reverse extraperitoneal tunneling to reach the ideal abdominal entry site without causing any extra injury.
ix. Resistance encountered during blunt tunneling through the rectus sheath can be easily overcome with a gentle rotating motion.
x. No other specialized instrument is needed during LERP technique, however, a custom-made low profile steel rod for tunneling is highly recommended.

## Conclusion

The LERP technique provides a fast and safe method to achieve reliable fixation of the distal catheter into the suprahepatic space.

## Supplementary Information

Below is the link to the electronic supplementary material.

ESM 1 Supplementary Material 1 (MP4 64.7 MB)

## Data Availability

No datasets were generated or analysed during the current study.

## Code availability

Not relevant.

## References

[1] Abdelmageed S, Sarkar P, Shlobin NA, Davila DG, Potts MB (2024) Laparoscopic-assisted peritoneal access in ventriculoperitoneal shunt placement: systematic review and meta-analysis. Neurosurgery 96(4):734–743. 10.1227/neu.0000000000003213 39465943 10.1227/neu.0000000000003213

[2] Ding Q, Wang J, Fan H, Jiang W, Guo H, Ji H, Song T, Xu S, Liu B (2023) Introduction and comparision of three different fixation methods in the suprahepatic space in laparoscopy-assisted ventriculoperitoneal shunt for hydrocephalus. Sci Rep 13:6231 10.1038/s41598-023-33566-5 37069252 10.1038/s41598-023-33566-5 PMC10110567

[3] Fernandez-Mendez R, Richards HK, Seeley HM, Pickard JD, Joannides AJ (2019) Current epidemiology of cerebrospinal fluid shunt surgery in the UK and Ireland (2004-2013). J Neurol Neurosurg Psychiatry 90:747–754 30910858 10.1136/jnnp-2018-319927 PMC6585267

[4] Habibi Z, Golpayegani M, Ashjaei B, Tayebi MK, Nejat F (2020) Suprahepatic space as an alternative site for distal catheter insertion in pseudocyst-associated ventriculoperitoneal shunt malfunction. J Neurosurg Pediatr 26(3):247–254. 10.3171/2020.3.PEDS19772 10.3171/2020.3.PEDS19772 32413860

[5] Hanak BW, Bonow RH, Harris CA, Browd SR (2017) Cerebrospinal fluid shunting complications in children. Pediatr Neurosurg 52:381–400 28249297 10.1159/000452840 PMC5915307

[6] Isaacs AM, Ball CG, Sader N, Muram S, Ben-Israel D, Urbaneja G, Dronyk J, Holubkov R, Hamilton MG (2021) Reducing the risks of proximal and distal shunt failure in adult hydrocephalus: a shunt outcomes quality improvement study. J Neurosurg. 10.3171/2021.2.jns202970 34450584 10.3171/2021.2.JNS202970

[7] Khalid SI, Nunna RS, Maasarani S, Shanker RM, Behbahani M, Edmondson CP, Mehta AI, Gupta SK, Chan EY, Torquati A, Byrne RW, Adogwa O (2021) Laparoscopic-assisted versus mini-open laparotomy for ventriculoperitoneal shunt placement in the Medicare population. Neurosurgery. 10.1093/neuros/nyaa541 33475722 10.1093/neuros/nyaa541

[8] Kim JH, Jung YJ, Chang CH (2016) Laparoscopic treatment of ventriculoperitoneal shunt complication caused by distal catheter isolation inside the falciform ligament. World Neurosurg 90:701–707 10.1016/j.wneu.2016.03.023 27001241

[9] Pandey A, Gangopadhyay AN, Sharma SP, Upadhyaya VD, Kumar V, Gopal SC, Gupta DK, Srivastava A (2009) Placement of the peritoneal end of a ventriculoperitoneal shunt in the suprahepatic space: does it improve prognosis? Pediatr Neurosurg 45:6–10 19221457 10.1159/000202618

[10] Svoboda SM, Park H, Naff N, Dorai Z, Williams MA, Youssef Y (2015) Preventing distal catheter obstruction in laparoscopic ventriculoperitoneal shunt placement in adults: the “falciform technique.” J Laparoendosc Adv Surg Tech A 25:642–645 26186206 10.1089/lap.2015.0196

